# Influence of Varied Waste Ceramic Fillers on the Resistance of Concrete to Freeze–Thaw Cycles

**DOI:** 10.3390/ma14030624

**Published:** 2021-01-29

**Authors:** Jacek Katzer, Jacek Halbiniak, Bogdan Langier, Maciej Major, Izabela Major

**Affiliations:** 1 Faculty of Geoengineering, University of Warmia and Mazury in Olsztyn, Heweliusza 4, 10-720 Olsztyn, Poland; jacek.katzer@uwm.edu.pl; 2 Faculty of Civil Engineering, Czestochowa University of Technology, Dabrowskiego 69, 42-201 Czestochowa, Poland; jacek.halbiniak@pcz.pl (J.H.); bogdan.langier@pcz.pl (B.L.); maciej.major@pcz.pl (M.M.)

**Keywords:** composite, concrete, recycling, ceramic waste, freeze–thaw resistance, pore characteristics

## Abstract

Our research focused on the influence of fillers obtained from crushed waste materials on the selected properties of concrete composites. The used waste materials were sourced from the production of ceramic tiles, ceramic pots, and sanitary ceramics. We evaluated concretes modified with the addition of 10% (by mass of cement) of different fillers. The properties, including the air content in the fresh concrete mix, consistency, compressive strength, and freeze-thaw resistance were examined. The evaluation of the freeze-thaw resistance was carried out by testing the concrete with the direct method for 150 cycles of freezing and thawing. The characteristics of the concrete porosity structure were assessed using automated digital image analysis. Concretes modified by coarse and fine fillers were characterized by different improvements in the mechanical properties and resistance to cycles of freezing and thawing. Composites with the addition of coarse fillers did not show any significant changes in comparison to the control concrete. An automated digital image analysis of the pore distribution in concrete proved to be an effective tool for the assessment of the freeze–thaw resistance of the concretes in question.

## 1. Introduction

Currently, a reduction of the volume of industrial waste and sustainable use of natural resources has become a necessity. The utilization of waste materials in the technology of mortars, concretes, or other cements and cement–like composites is one of key methods of environmental protection. This offers opportunities for the recycling and utilization of significant amounts of different types of waste materials [1]. In certain sectors of ceramics production, the amount of waste may reach up to 30% [2]. Numerous studies have examined concretes [3,4,5,6,7,8,9,10] containing various types of ceramic waste (obtained from construction elements [11,12,13], sanitary products [14], ceramic electric insulators [15], porcelain pottery [16], etc.).

The general conclusion of the research in question was that concretes with the addition of ceramic fillers have good workability and strength characteristics, comparable to ordinary concretes. In the research carried out by Siddique et al. [17], the research team used a fine aggregate from recycled porcelain pottery as an additive for concrete. During the study, 20% to 100% of natural sand was replaced by the porcelain pottery waste. The strength characteristics of the achieved concrete were improved in comparison to the reference concrete. One of key elements of the study in question was the pore analysis of the achieved concrete. They established that an increase in the pore content did not lead to the deterioration of the freeze-thaw resistance.

The durability of concrete exposed to freeze-thaw cycles is not directly affected by the porosity but by the size of pores and their spacing [18]. The uniform spacing of pores of specific diameters in hardened concrete enables the creation of durable concrete structures that are resistant to harsh environmental conditions. Harnessing ceramic waste in the form of fine or coarse fillers can have a beneficial effect on the pore structure in concrete. Examinations of the freeze-thaw resistance of concrete modified by the addition of meal obtained from crushed ceramic pots were described by Halbiniak et al. [19]. The concrete with the addition of the pulverized ceramic material in the amounts of 10%, 20%, and 30% of the cement mass was characterized by higher compressive strength, lower water absorption, and lower values of the depth of water penetration in comparison to the reference concrete.

Taking all the above facts into consideration, we decided to conduct research dedicated to studying the influence of varied waste ceramic fillers on the aeration and mechanical characteristics of concrete. We hypothesized that the addition of specific fine or coarse ceramic filler could shape the internal pore structure of concrete and transform it into a highly freeze-thaw resistant material. One must keep in mind that ceramic waste (both white and red) is commonly available. Up to 30% of the pottery that is produced is crushed, damaged, or dismissed during production [19]. Building rubble from the demolition of degraded buildings and ceramic elements (both wall and roof) dismissed during the production process are the main source of waste red ceramics.

## 2. Materials

Ordinary tap water and Portland cement CEM I 42.5R (CEMEX Polska, Rudniki, Poland) were used for the creation of all planned concrete mixes. The tap water characteristics were presented in a previous publication [19]. The chemical composition and other properties of the used cement (as given by producer) are presented in Table 1.

**Figure 1:**
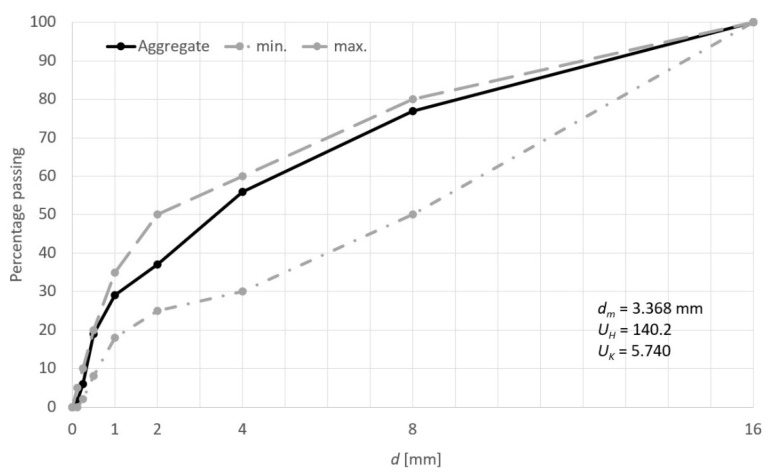
Grading curve of the used natural aggregates blend with the grading requirements.

A mix of natural aggregates was used as the main aggregate. To obtain the optimal granular composition of the natural aggregate, we experimentally composed three aggregates (sand, fine gravel 2–8 mm, and coarse gravel 8–16 mm). The sieve analysis of the aggregate mix was conducted in accordance with the requirements of the standard EN 933–1:2012 [20]. The achieved grading curve of the natural aggregates blend is presented in Figure 1 together with the grading requirements (min. and max. limits according to PN–EN 206–1:2003).

**Figure 2:**
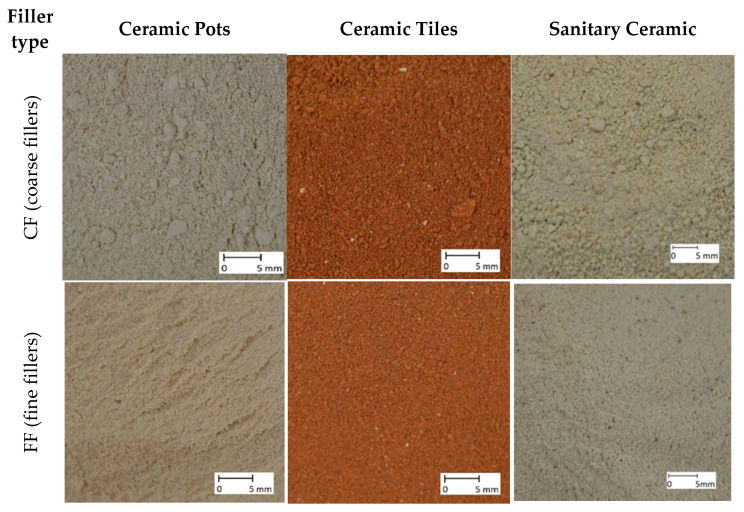
Optical microscope images of the used ceramic fillers of different origin.

Three different ceramic wastes were utilized in this research-namely waste from the production of ceramic pots, ceramic tiles, and sanitary ceramics was used for the creation of ceramic fillers. The procedure of the filler creation consisted of two stages. First, ceramic waste was ground using a basic ball mill. In this way, coarse fillers (CF) were obtained, characterized by a maximum particle diameter of 4 mm. Secondly, amounts of CFs were fed to a disintegrator. As a result of processing with the disintegrator, fine fillers (FF) were achieved. The maximum size of the particle diameter of all FFs was 0.5 mm. The ball mill used, disintegrator, and the procedure of the preparation of ceramic fillers were thoroughly described in previous publications [19,21]. The granular compositions of all prepared fine and coarse fillers are presented in Table 2. Optical microscope images of the fillers are presented in Figure 2.

All mixes were modified by a commercially available superplasticizer based on sulfonated naphthalene polycondensate (lignosulfonate). This was characterized by a density of 1.155 ± 0.020 kg/dm^3^, pH equal to 5.0 ± 1.0, total chloride ion content less than 0.1%, and sodium oxide equivalent less than 2.0%. The admixture was dosed in the amount of 1.0% of the cement weight. This is a moderate volume of dosage as recommended by the producer.

## 3. Research Program

All tested mixes were prepared harnessing the same procedure. First, the dry ingredients (natural aggregate, cement, and ceramic filler) were added to a mixer. They were mixed for 10 s. Subsequently, water with the diluted admixture was added to the mixer. All ingredients were mixed for 90 s. Ceramic fillers were utilized to partially substitute for the aggregate. Taking into account the previous experience of the research team with similar waste materials [19,22,23,24], we decided to substitute 10% (by weight) of the aggregate with ceramic fillers. The density of the natural aggregate, ceramic pots, ceramic tiles, and sanitary ceramics was equal to 2.61, 2.40, 2.65, and 2.52 kg/dm^3^, respectively. The compositions of the cast mixes are summarized in Table 3.

During the research program, the natural aggregate was replaced with ceramic fillers by mass (0.54%). The volume of the created mixes was not constant; however, the differences were negligible. The volumes of the mixes were equal to 999.8, 1000.0, 1000.5, and 1001.2 dm^3^ for concrete with ceramic tiles, no filler, sanitary ceramics, and ceramic pots, respectively. This indicates that the mixes were differentiated up to 0.12% of the volume in comparison to the reference concrete. We concisely adopted this approach (which does not influence the key results in any noticeable way) due to our experience with similar mixes as described in [19].

**Figure 3:**
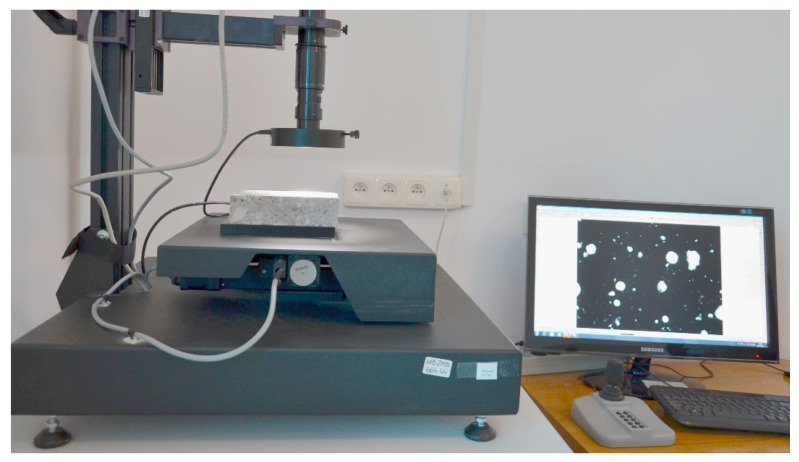
Lab set-up for testing the porosity structure of the hardened concrete.

The conducted research consisted of three main stages. The first stage was focused on testing the properties of the fresh concrete mix. During this part of the research, the consistency of the fresh mix and air content (Ac) were evaluated. For the consistency test, an ordinary slump procedure according to EN 12350–2:2011 [25] was harnessed. The air content in the fresh mix was tested using the methodology described in EN 12350–7:2011 [26]. The second stage was focused on non–destructive tests, such as SEM-EDX (LEO Electron Microscopy Ltd, England) evaluation of the elemental composition and evaluation of the air pore distribution in hardened concrete according to EN 480–11:2008 [27] using image analysis [28,29,30]. An apparatus with a high quality digital camera and dedicated image analyzing software was utilized for this task [28,29,30]. The harnessed lab set-up is presented in Figure 3.

The third stage of the research was dedicated to destructive tests. The compressive strength after 28 days of curing was tested according to EN 12390–3:2011 [31] using three cubed specimens (150 mm × 150 mm × 150 mm) from each batch. The freeze–thaw resistance was tested (according to [32]) using twelve cube specimens (10 cm × 10 cm × 10 cm) from each batch. Six specimens were tested after 150 cycles of freeze-thaw, and six served as a reference point. The specimens prepared for these tests after 28 days of curing were saturated for 7 days to ensure full saturation. The subsequent freeze-thaw cycles lasted for 50 days. Thus, the strength tests of both the reference specimens and specimens exposed to freeze-thaw cycles took place 85 days after casting.

## 4. Results

The achieved properties of the fresh concrete mixes are presented in Table 4. The consistency of the mixes ranged from 40 to 55 mm. The achieved results were on the edge between consistency class S1 and S2 according to [25].

**Figure 4:**
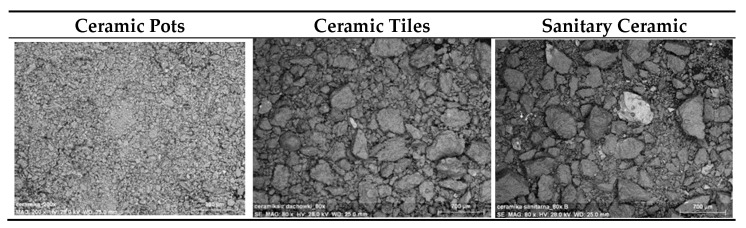
SEM images of the used ceramic fine fillers obtained from ceramic pots, ceramic tiles and sanitary ceramic.

SEM images of the used ceramic fine fillers are presented in Figure 4. The results of the SEM analysis of the fillers are presented in Table 5 as an Elemental composition.

**Figure 5:**
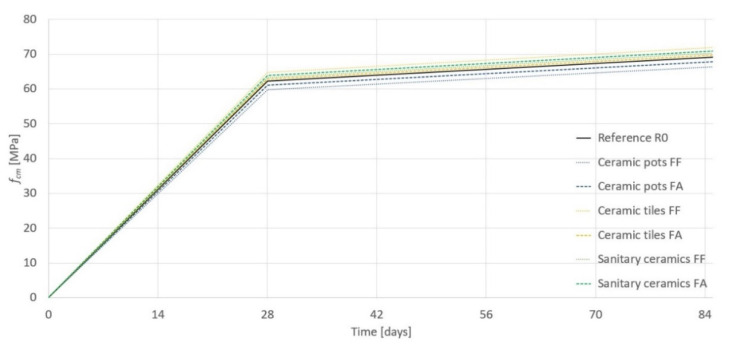
The development of the compressive strength over time.

The results of the compressive strength test (after 28 days, conducted on cube specimens 15 cm × 15 cm × 15 cm) of the reference concrete and the concretes modified by ceramic fillers are presented in Table 6 together with the strength loss after 150 freeze-thaw cycles. The development of the compressive strength over time for the concretes in question is shown in Figure 5.

The results of the image analysis of the cross–sections of hardened concretes are presented in Table 7. The total air content in concrete *A*, pore distribution index $\bar{L}$ [33], and the content of micro-pores *A*_300_ [33] were determined. While analyzing the results presented in Table 7, one should keep in mind that desired values of $\bar{L}$ and *A*_300_ should be up to 0.20 mm and over 1.5%, respectively [28,29,33].

## 5. Discussion

The most favorable effect of the ceramic filler on the compressive strength was found in the case of using ceramic tiles FF and sanitary ceramics CF. The influence of all examined ceramic fillers on compressive strength was relatively small and reached up to 4%. This increase in compressive strength was negligible from the point of view of the strength class, which would be granted to the concretes in question. All tested concretes would be described by the strength class C 45/55 according to PN–EN 206:2014.

Ceramic pots were characterized by a higher Al content compared with ceramic tiles and sanitary ceramics. Ceramic pots contain significantly less Fe in comparison to sanitary pots and ceramic tiles (0.97%, 11.55%, and 15.01%, respectively). The amounts of Na and K are similar in all three ceramics types in question. Taking into account the development of the compressive strength of the tested concretes (see Figure 5), the elemental composition of the different ceramics did not affect it. The possibility of a reaction between the ceramics fillers and cement paste should be investigated due to the amounts of Na and K.

The resistance to freeze–thaw cycles was improved for each modified concrete. All used ceramic fillers reduced the loss of compressive strength after 150 freeze–thaw cycles. The loss ranged from 5.3% to 15.2%. The delamination process was clearly slowed down. 

The porosity of hardened concrete and the pore diameter distribution were also significantly influenced by the addition of ceramic fillers. The content of the micro–pores *A*_300_ ranged from 1.2% for the reference concrete to 2.3% for the concrete modified by the fine filler obtained from ceramic tiles. The second largest content of the micro–pores *A*_300_ was achieved by concretes modified by fine filler obtained from ceramic pots and sanitary ceramics. Both contents of micro–pores *A*_300_ values were equal to 2.0% for those concretes. 

All concretes with ceramic fillers except CF were characterized by *A*_300_ > 1.5%, which was recognized by multiple researchers [28,29,33] as the threshold for achieving concrete resistant to freeze–thaw cycles. The resistance to freeze–thaw cycles stayed in a direct correlation with the type of used ceramic filler. The best effects were achieved by the addition of fine fillers, which lowered the diameter of the pores, thus, increasing the resistance to freeze–thaw cycles. The addition of coarse fillers was less successful. The resistance to freeze–thaw cycles of concretes modified by coarse fillers was lowered by up to 5.5 percentage points in comparison to the reference concrete.

## 6. Conclusions

The results obtained in the study lead to the following conclusions:

- The effect of the addition of ceramic fillers (both fine and coarse) on the compressive strength was negligible.
- The addition of ceramic fillers increased the aeration of concrete.
- Concretes with ceramic fillers were characterized by higher contents of micro–pores in class 18 (with a diameter of up to 300 µm) in comparison to the reference concrete.
- The content of micro–pores directly influenced the resistance of the tested concretes to freeze–thaw cycles.
- The best effect on the resistance to freeze–thaw cycles was achieved by the addition of all three fine fillers that were considered in this study.
- Additional tests should be conducted using different ceramic fillers to confirm the achieved results.
- The possibility of reactions between the fillers and cement paste should be investigated in the future.

## Figures and Tables

**Table 1:** The Portland cement CEM I 42.5R characteristics.

Parameter	Unit	Value
Chemical composition		
CaO	%	62.5
SiO_2_	%	20.3
Al_2_O_3_	%	5.0
Fe_2_O_3_	%	3.3
MgO	%	1.8
SO3	%	3.0
Cl	%	< 0.1
Setting time		
Initial	min.	128
Final	min.	163
Specific Surface Area (Blaine)	cm^2^/g	4604
Compressive strength		
After 2 days	Mpa	25.7
After 28 days	Mpa	58.8

**Table 2:** The granular composition of the used ceramic fillers. Coarse fillers (CF) and fine fillers (FF).

Fraction (mm)	Ceramics Pots		Ceramics Tiles		Sanitary Ceramics	
	CF	FF	CF	FF	CF	FF
0–0.063	1	30	7	31	3	32
0.063–0.125	2	29	6	34	3	36
0.125–0.25	18	36	35	32	17	28
0.25–0.5	25	5	22	3	12	4
0.5–1	30	-	18	-	22	-
1–2	20	-	10	-	31	-
2–4	4	-	2	-	12	-
*d_m_* (mm)	0.567	0.106	0.273	0.096	0.841	0.092

**Table 3:** The compositions of the tested concrete mixes.

Concrete Mix	Component Content (kg/m^3^)					
	CF	FF	Aggregate	Water	Cement	Admixture
Reference R0	-	-	1920			
Ceramic Pots PF	-	35.5				
Ceramic Pots PA	35.5	-				
Ceramic Tiles TF	-	35.5	1885	149	355	3.55
Ceramic Tiles TA	35.5	-				
Sanitary Ceramics SF	-	35.5				
Sanitary Ceramics SA	35.5	-				

**Table 4:** The properties of the fresh mixes.

Property	Reference	Ceramics Pots		Ceramics Tiles		Sanitary Ceramics	
	R0	FF	CF	FF	CF	FF	CF
*A_c_* (%)	2.4	5.5	2.7	4.0	2.6	3.9	2.8
*h_slump_* (mm)	45	40	50	55	40	55	45
Consistency class	S1	S1	S2	S2	S1	S2	S1

**Table 5:** Elemental composition of ceramics obtained by the means of SEM/EDX method.

Spectrum	Element Content (%) by Mass		
	Ceramics Pots	Ceramics Tiles	Sanitary Ceramics
C	0.27	0.19	0.13
O	40.79	36.36	36.71
Na	1.25	1.55	1.41
Mg	0.58	1.95	-
Al	17.38	10.32	13.19
Si	35.37	27.21	30.32
P	-	0.19	0.05
S	-	0.03	-
K	2.63	2.29	2.30
Ca	0.30	2.40	1.53
Ti	0.48	0.93	0.28
Mn	-	0.84	-
Fe	0.97	15.01	11.55
Zn	-	0.22	0.73
Zr	-	-	1.25
Ba	-	-	0.55

**Table 6:** The properties of the hardened concretes.

Property	Reference	Ceramics Pots		Ceramics Tiles		Sanitary Ceramics	
	R0	FF	CF	FF	CF	FF	CF
*f_cm_* (MPa)	62.2	59.7	61.1	64.7	62.8	63.3	63.8
Δ f_cm150_ after f–t cycles (%)	9.7	5.3	14.3	6.5	11.9	7.0	15.2

**Table 7:** The characteristics of the air pores in the hardened concrete.

Property	Reference	Ceramics Pots		Ceramics Tiles		Sanitary Ceramics	
	R0	FF	CF	FF	CF	FF	CF
*A* (%)	2.6	4.8	3.5	3.6	3.3	3.3	3.0
$\bar{L}$ (mm)	0.2	0.2	0.2	0.16	0.23	0.24	0.28
*A*_300_ (%)	1.2	2.0	1.3	2.3	1.6	2.0	1.6

## Data Availability

The data presented in this study is available within the article.
